# Dissecting the Metabolic Phenotype of the Antihypertensive Effects of Five *Uncaria* Species on Spontaneously Hypertensive Rats

**DOI:** 10.3389/fphar.2019.00845

**Published:** 2019-07-30

**Authors:** Zijin Feng, Jinjun Hou, Yang Yu, Wenyong Wu, Yanping Deng, Xia Wang, Haijuan Zhi, Linlin Zhang, Wanying Wu, De-an Guo

**Affiliations:** ^1^College of Traditional Chinese Medicine, China Pharmaceutical University, Nanjing, China; ^2^Shanghai Research Center for Modernization of Traditional Chinese Medicine, National Engineering Laboratory for TCM Standardization Technology, Shanghai Institute of Materia Medica, Chinese Academy of Sciences, Shanghai, China

**Keywords:** Uncaria Stem with Hooks, multiple botanical origins, spontaneously hypertensive rats, systolic blood pressure, metabonomics, indole alkaloids

## Abstract

The sourcing of plants from multiple botanical origins is a common phenomenon in traditional Chinese medicines. *Uncaria* Stem with Hooks (UHs) are approved for using five botanical origins in the Chinese Pharmacopoeia (2015 Edition). All five UHs are commonly used for treating hypertension even though the plants have different chromatographic fingerprints based on our previous study. However, their hypotensive effects and metabolic phenotypes have not been fully studied. In the present study, spontaneously hypertensive rats (SHRs) were orally administered five aqueous extracts (4 g crude drug/kg) prepared from the different UHs over a 6-week period. Systolic blood pressure (SBP) was measured every week, and urine was collected after SBP measurement. Untargeted metabonomics was performed using ultra performance liquid chromatography (UPLC) coupled with an LTQ-Orbitrap mass spectrometer. Bidirectional orthogonal projection to latent structures discriminant analysis (O2PLS-DA), Student’s *t* test, and correlation analysis were used for pattern recognition and the selection of significant metabolites. A similar and prolonged reduction in SBP was observed in each of the groups given the five different UHs, while the metabolic profiles were perturbed slightly compared with that of SHR. Further analysis has shown that only a few common, different components were observed within the five groups, which showed the similar antihypertensive effect in spite of the distinct metabolic pathways due to their different alkaloid composition. These results help in understanding the mechanisms of the phenomenon “different species, same effect” of UHs.

## Introduction

Traditional Chinese medicines (TCMs) are important alternative therapeutics for chronic diseases because of their significant biological effects in disease prevention and treatment (Yim et al., 2000; Chen et al., 2008; Lee et al., 2013). TCMs include natural materials originating from botanical, mineral, and animal sources, in which botanical materials dominate, representing about 87% of the sourced materials (Chinese Pharmacopoeia Commission, 2010). In the botanical materials, the phenomenon of multiple botanical origins (one-to-multiple) is common. It means that one recommended constituent in a remedy may be derived from multiple botanical species, instead of assigned individual plant for the remedy (Wu et al., 2007; Tian et al., 2009; Da et al., 2012). TCM materials derived from different plant species usually differ in morphology, as well as their microscopic and chemical characteristics. However, they can have similar clinical applications and effects (Zheng et al., 2010; Ashfaq et al., 2011). We call this phenomenon “different species, same effect.” Approximately 25.4% of crude drugs described in the Chinese Pharmacopoeia (2015 Edition) have two or more approved origins (Hou et al., 2018). The existence of “one-to-multiple” substituting medicinal plants leads to the quality control and clinical efficacy issues in the manufacture of TCM products and their clinical use. For patients and practitioners anticipating assurances of effectiveness, this situation enhances the importance of assessing the medicinal plant species and conducting respective metabolite analysis. Therefore, it is urgent to understand how similar clinical effects could be produced by TCMs with quite different chemical profiles.

Uncaria Stem with Hooks (UHs) is one example of this type of TCM with five designated botanical origins, named *Uncaria rhynchophylla* (Miq). Miq. ex Havil. (URM), *Uncaria macrophylla* Wall. (UMW), *Uncaria sinensis* (Oliv). Havil. (USH), *Uncaria hirsuta* Havil. (UHH), and *Uncaria sessilifructus* Roxb. (USR) (National Commission of Chinese Pharmacopoeia, 2015). UHs were employed in treating hypertension as a safe and effective therapy based on having characteristics of multiple biological targets, multiple functional pathways, and fewer side effects (Feng et al., 2012). As a complex, multifactorial disease, hypertension is closely associated with genetic, environmental, and metabolic factors, being the most vital risk factor for cardiovascular and chronic kidney diseases (Perry et al., 1995). Indole alkaloids are recognized as the active pharmacological components of UHs (Chen et al., 2010; Zhou and Zhou, 2012; Yasushi et al., 2018). Our previous research has also shown that the methanol extract of five UHs had very different chromatographic fingerprints using LC-MS (Pan et al., 2015; Pan et al., 2017; Hou et al., 2018; Pan et al., 2018). For *Uncaria rhynchophylla* (Miq). Miq. ex Havil.(URM), it mainly contained tetracyclic monoterpenoid indole alkaloids, tetracyclic monoterpenoid oxindole alkaloids, and glycosidic alkaloids, while for UMW and USR, although their fingerprints were different, they had similar components composed of tetracyclic monoterpenoid oxindole alkaloids and tetracyclic monoterpenoid indole alkaloid *N*-oxides. Similarly, for USH and UHH, pentacyclic monoterpenoid oxindole alkaloids and their derivatives were mainly found in these samples. Therefore, the antihypertensive action and mechanisms of the five different UHs are yet to be explored.

As an important branch of systems biology, metabonomics provides a comprehensive insight into the role of metabolite changes at chemical and biological levels. The metabolic variations of an organism can be systematically explored using the results of comprehensive metabolite analysis (Nicholson et al., 1999). Metabonomics has been widely applied in exploring the pathological mechanisms of cardiovascular-related disorders (Kordalewska and Markuszewski, 2015), including coronary heart disease, hypertension, stroke, heart failure, atrial fibrillation, etc. In addition, metabonomics has shown important advantages as an approach to identify endogenous metabolites that relate to drug efficacy (Yang et al., 2012). Furthermore, metabonomics is widely used to understand the therapeutic mechanisms of action of medicinal plants in a variety of cardiovascular diseases. These advantages of metabonomics make it a preferred method to study the mechanisms of TCMs, which is consistent with the holistic and systemic properties of TCMs. Metabonomics analysis requires high-precision, high-density information on a wide range of compound classes and chemical profiles. Many chromatographic and spectroscopic technologies, including NMR, GC-MS, LC-MS, and CE-MS (Lindon and Nicholson, 2008), have been applied to perform metabonomics analysis. Because of its accuracy, reproducibility, and high sensitivity, LC-MS became the most frequently applied and significant hyphenated tools in metabonomics research (Liu et al., 2011).

In this study, the antihypertensive effects of the five different UHs on spontaneously hypertensive rats (SHRs) were examined after repeated oral administration. In addition, the metabolite profile in the urine of the SHRs was analyzed by UPLC-LTQ-Orbitrap-MS. The antihypertensive efficacy and the metabolic phenotypes of the UHs were compared. An explorative study was also performed to identify potential biomarkers for hypertension with or without UHs treatment. These results provide additional evidence to understand the phenomenon “different species, same effect.”

## Materials and Methods

### Chemicals and Reagents

High performance liquid chromatography (HPLC)-grade acetonitrile and methanol were acquired from Merck KGaA (Merck, Darmstadt, Germany), and formic acid and ammonia from Sigma-Aldrich (St. Louis, MO, USA) were used as the mobile phase. Deionized water (18.2 MΩ cm at 25°C) was prepared using a Millipore Alpha-Q water purification system (Millipore, Bedford, USA). The five UHs plant materials, *Uncaria rhynchophylla* (Miq). Miq. ex Havil. (URM), *Uncaria macrophylla* Wall. (UMW), *Uncaria sinensis* (Oliv). Havil. (USH), *Uncaria hirsuta* Havil. (UHH), and *Uncaria sessilifructus* Roxb. (USR), were kindly donated by Dr. Huiqin Pan. Their authentication was based on the botanical traits recorded in the Chinese Flora (http://frps.iplant.cn/frps/Uncaria). The voucher specimens from the five species were deposited at the herbarium of the Shanghai Research Center for Modernization of Traditional Chinese Medicine.

### Sample Preparation and Analysis

Samples of the five different UHs (1 kg) were ground and passed through a No. 2 sieve (850 µm). The passing rate of the particles was maintained at more than 80%. The powders were extracted twice by refluxing with boiling water (1:15, w/v) for 20 min each time. The combined solution obtained was concentrated under reduced pressure and the aqueous extract lyophilized using a freezing vacuum dryer.

The freeze-dried powders of the five different water extracts (0.1 g) were dissolved in distilled water (50 ml) in a covered 100-ml conical flask. After centrifugation at 20,817×*g* for 10 min, the supernatant was analyzed using LC-HRMS. The detailed analytical methods are presented in the Supporting Information.

### Animals and Dosing

The animal studies were carried out after approval of the protocol by the Animal Ethics Committee of Shanghai Institute of Materia Medica (Shanghai, China). The protocol number was 2017-10-GDA-47. Male SHRs and normotensive control Wistar Kyoto rats (WKYs), 10 weeks old, were purchased from Wei Tong Li Hua Animal Center (Beijing, China). Rats were housed individually in metabolic cages in a standard animal laboratory with a 12-h light/dark cycle. Water and standard rat chow were available *ad libitum*, and the rats were acclimated to the facilities and environment for 2 weeks before the experiments were initiated.

The rats were then randomly divided into eight groups (*n* = 10). The following preparations were administered to the rats based on the groups to which they were assigned: vehicle (deionized water) or agents suspended in the vehicle, including captopril at 30 mg/kg (i.g. per day), *Uncaria rhynchophylla* (Miq). Miq. ex Havil. (URM), *Uncaria macrophylla* Wall. (UMW), *Uncaria sinensis* (Oliv.) Havil. (USH), *Uncaria hirsuta* Havil. (UHH), and *Uncaria sessilifructus* Roxb. (USR) at 4.00 g/kg (measured as the quantity of crude material administered i.g. per day, the dose was calculated as human dose 12 g/60 kg/day). All animals were given the gastric infusion once at same time of day for a continuous period of 6 weeks.

### Measurement of SBP and Collection of Sample

The systolic blood pressure (SBP) of the rats in a conscious state was measured (Shanghai Alcott Biotech Co., Ltd, China) once a week after dosing 1 h, using the indirect plethysmographic tail-cuff method before urine sampling. After 12 h administration, each rat was placed into an individual metabolic cage to collect urine for 12 h, and the acquired urine samples were stored at −80°C.

### Histopathology Analysis

At the end of the experiment, the rats were killed through exsanguination (abdominal aortic artery) under isoflurane anesthesia. The heart and arch of the aorta were quickly removed and fixed in 10% formalin for histopathological examination. The tissues were then further processed, embedded in paraffin, and stained with hematoxylin and eosin.

### Urine Sample Handling

Urine samples were centrifuged at 20,817×*g* for 10 min at 4°C and diluted in water. The dilution factors were estimated by the creatinine peak areas of each urine sample. Creatinine analysis was carried out in this laboratory using an HPLC procedure (Ministry of Health of the People's Republic of China).

### Chromatography and Mass Spectrometry Conditions

High-resolution mass spectra for metabolomic analysis were obtained on an LTQ-Orbitrap Velos Pro hybrid mass spectrometer (Thermo Fisher Scientific, San Jose, CA, USA) connected to an Ultimate 3000 UHPLC system. The separation of samples was performed on a Waters ACQUITY UPLC HSS T_3_ column (1.8 µm, 2.1 mm × 100 mm) with an online filter. The mobile phase was comprised of solvent A [100% H_2_O (0.1% formic acid)] and solvent B [100% acetonitrile (0.1% formic acid)], with gradient elution as follows: 99% A at 0–1 min, 99–85% A at 1–3 min, 85–50% A at 3–6 min, 50–5% A at 6–9 min, and 5% A at 9–10 min. The flowrate was kept at 0.50 ml/min. The temperatures of the autosampler and column were kept at 4 and 40°C, respectively. The injection volume of all samples was set at 5.0 µl. Positive and negative ion detection modes were conducted in these analyses. The electospray ionization (ESI) source parameters were set as follows: ion spray voltage, 3.5 kV for positive ion mode and 2.7 kV for negative ion mode; capillary temperature, 380°C; source heater temperature, 350°C; sheath gas (N_2_), 60 arbitrary units; auxiliary gas (N_2_), 50 arbitrary units; and sweep gas (N_2_), 10 arbitrary units. The Orbitrap analyzer scanned the masses within the range of *m*/*z* 50–1,200 and at a resolution of 30,000 for MS. Samples were analyzed randomly for unbiased measurement with a deuterated reference solution as internal standards to ensure accuracy and reproducibility.

### Data Processing and Pattern Recognition Analysis

The LC-MS raw data were processed using the Qualbrowser module of Xcalibur version 2.2 (Thermo Fisher Scientific, Inc., Waltham, MA, USA). The peak alignment, peak picking, and normalization routines were performed using Progenesis QI 3.3.1 (Waters, USA) to obtain the data matrix. Preprocessed data were then exported as a .csv file for further multivariate data analysis.

Before chemometric analysis, the missing values for each sample class were treated using the 80% rule. The data were introduced to SIMCA-P V14.0 (Umetrics, Sweden, Stockholm) for orthogonal partial least squares discriminant analysis (OPLS-DA) and bidirectional orthogonal projection to latent structures discriminant analysis (O2PLS-DA). The quality of the models was evaluated with the relevant *R* ^2^ and *Q* ^2^. The statistical significance was calculated using the Student’s *t* test (*p* < 0.05), as implemented in the Microsoft Office Excel 2017 software.

### Identification of Biomarkers and Metabolic Pathway Analysis

For the identification of potential biomarkers, the accurate MS fragments of the metabolites were searched using OSI/SMMS software (One-Step Solution for Identification of Small Molecules in Metabonomics Studies) and online free databases, such as the Human Metabolome Database (http://www.hmdb.ca/) and Metlin (http://metlin.scripps.edu/). Pathway analysis was performed by KEGG (http://www.genome.jp/kegg/pathway.html), which is a useful web-based tool for pathway analysis and visualization of metabonomics data.

## Results

### Analysis of the Aqueous Extracts of the Five Different UHs

**Figure 1:**
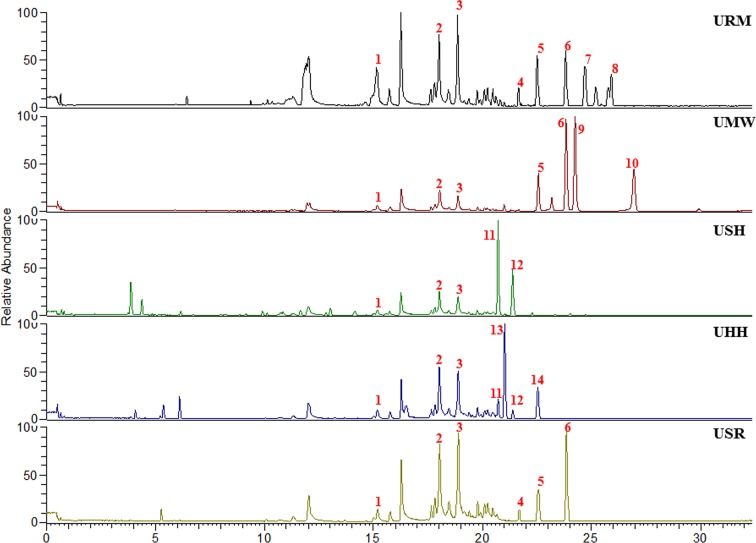
The HPLC fingerprints of five different Uncaria Stem with Hooks (UHs) species.

The chromatographic fingerprints of the aqueous extracts from the five different UHs are shown in **Figure 1**. Through comparison with our established compound library of the five different UHs (Pan et al., 2018), 14 peaks were identified (**Table 1**). Among them, compounds 1–3 were common ingredients in all five different UHs. Compounds 4–6 were mainly found in the extracts of *Uncaria rhynchophylla* (Miq). Miq. ex Havil. (URM), *Uncaria macrophylla* Wall. (UMW), and *Uncaria sessilifructus* Roxb. (USR). Compounds 11 and 12 were present in the samples of *Uncaria sinensis* (Oliv). Havil. (USH) and *Uncaria hirsuta* Havil. (UHH). There were also some distinctive compounds in the different UHs, such as compounds 7 and 8 in *Uncaria rhynchophylla* (Miq). Miq. ex Havil. (URM), 9 and 10 in *Uncaria macrophylla* Wall. (UMW), and 13 and 14 in *Uncaria hirsuta* Havil. (UHH). These results confirmed that the chromatographic fingerprints and the main components of the five individual *Uncaria* species were quite different.

**Table 1:** U-HPLC/LTQ-Orbitrap analysis of the aqueous extract of the Uncaria Stem with Hooks (UHs).

No.	tR/min	*m*/*z*	MS^n^ fragment ions	Identification
1	16.26	499.2031	MS^2^:337.16, 267.11, 319.15; MS^3^:171.09, 144.08	Vincosamide
2	18.01	535.2632	MS^2^:373.21, 355.20, 323.18, 281.05; MS^3^:160.08, 214.14	16,17-Dihydro-*O*-demethylrhynchophylline-glc or isomer
3	18.84	535.2635	MS^2^:373.21, 355.20, 323.18; MS^3^:160.08, 214.15, 269.17	16,17-Dihydro-*O*-demethylrhynchophylline-glc or isomer
4	21.63	383.1956	MS^2^:351.17, 267.15, 319.15, 201.10; MS^3^:160.08, 267.15, 269.17	Isocorynoxeine
5	22.48	385.2104	MS^2^:353.19, 267.15, 269.17, 160.08; MS^3^:241.13, 187.09, 213.10	Isorhynchophylline
6	23.79	385.2111	MS^2^:353.19, 269.17, 267.15, 215.12; MS^3^:160.08, 213.10, 241.13	Rhynchophylline
7	24.68	367.2005	MS^2^:224.13, 236.13, 335.18, 298.15; MS^3^:108.08, 192.10	Hirsuteine
8	25.88	369.2156	MS^2^:226.14, 238.14, 337.19, 352.19, 298.14; MS^3^:110.10	Hirsutine
9	24.21	385.2106	MS^2^:353.19, 269.17, 267.15; MS^3^:241.13, 160.08, 187.09, 213.10	Corynoxine B
10	26.91	385.2105	MS^2^:353.19, 267.15, 241.13; MS^3^:241.13, 187.09, 160.08, 265.13	Corynoxine
11	20.72	369.1792	MS^2^:337.16, 309.16, 241.13; MS^3^:158.06, 201.10, 160.08, 213.10	Isomitraphylline
12	21.38	369.1794	MS^2^:337.16, 309.16; MS^3^:160.08, 158.06, 201.10, 213.10, 187.09	Mitraphylline
13	21.01	369.1795	MS^2^:337.16, 309.16, 201.10; MS^3^:160.08, 158.06, 201.10, 309.16	Uncarine B
14	22.54	369.1792	MS^2^:337.16, 309.16, 281.09; MS^3^:158.06, 160.08, 201.10, 265.10	Uncarine A

### Systolic Blood Pressure (SBP) of SHR Treated With Captopril, URM, UMW, UHH, USH, and USR

**Figure 2:**
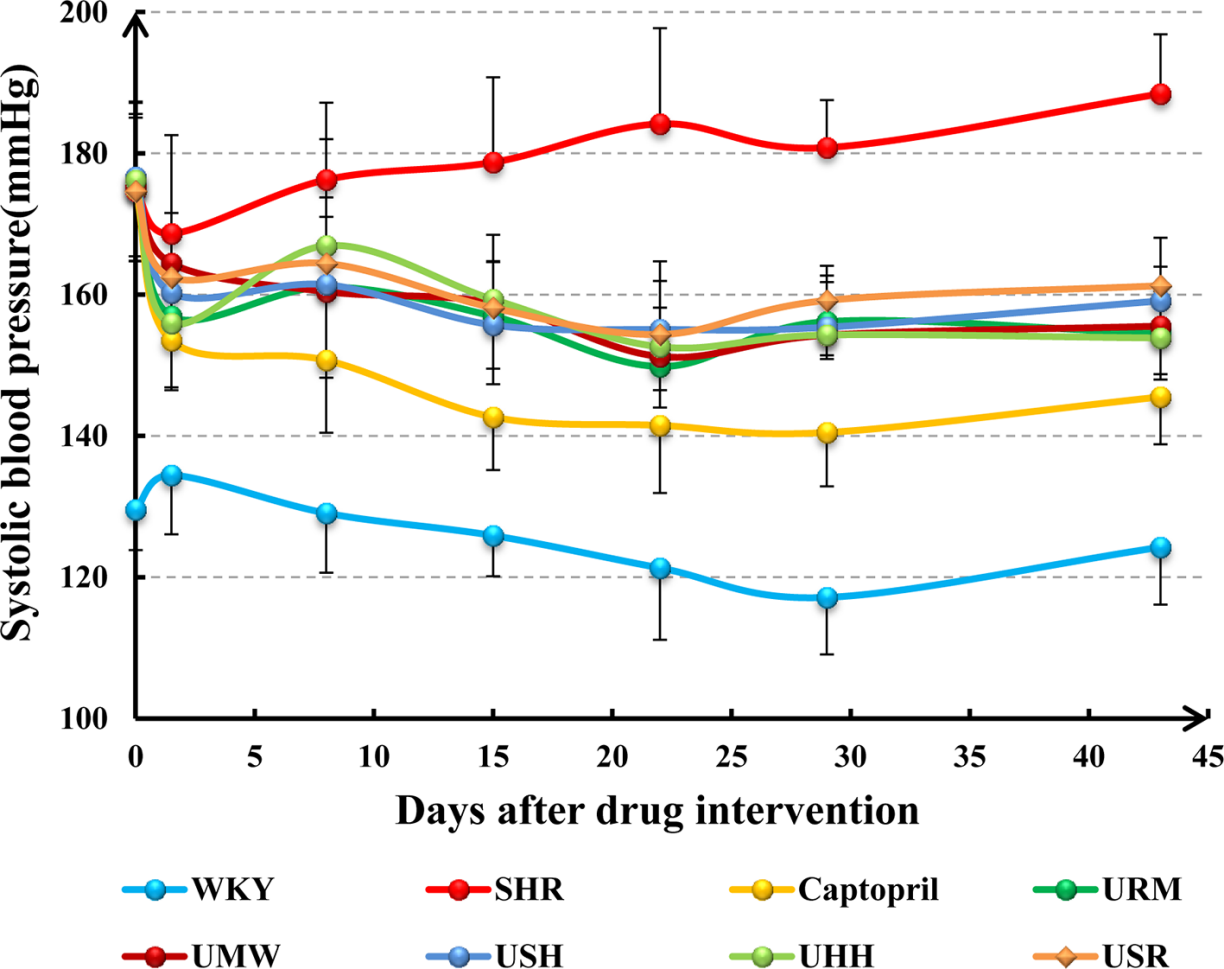
Systolic blood pressure (SBP) measured from 0 to 43 days of treatment with captopril, URM, UMW, USH, UHH, and USR.

The systolic blood pressure (SBP) of the control group (WKY) remained stable during the 6-week time course (**Figure 2**). The SBP of the SHR model group increased gradually from day 1 to 43, and their SBP remained steady at a high level, 20–40% higher than that of the normotensive WKY rats. The SBP of the animals was measured once a week after treatment with captopril, URM, UMW, UHH, USH, or USR. The SBP decreased rapidly in all of the treated groups after the first treatment. As the treatments continued, the SBP continued to decrease slowly in all groups. Six weeks later, treatments were stopped, and all UHs-treated groups remained at a lower SBP than the SHR model groups (*p* < 0.05). These results demonstrated that all five different UHs had similar effects on decreasing SBP.

### Histopathological Evaluation of UHs’ Cardioprotective Effects

**Figure 3:**
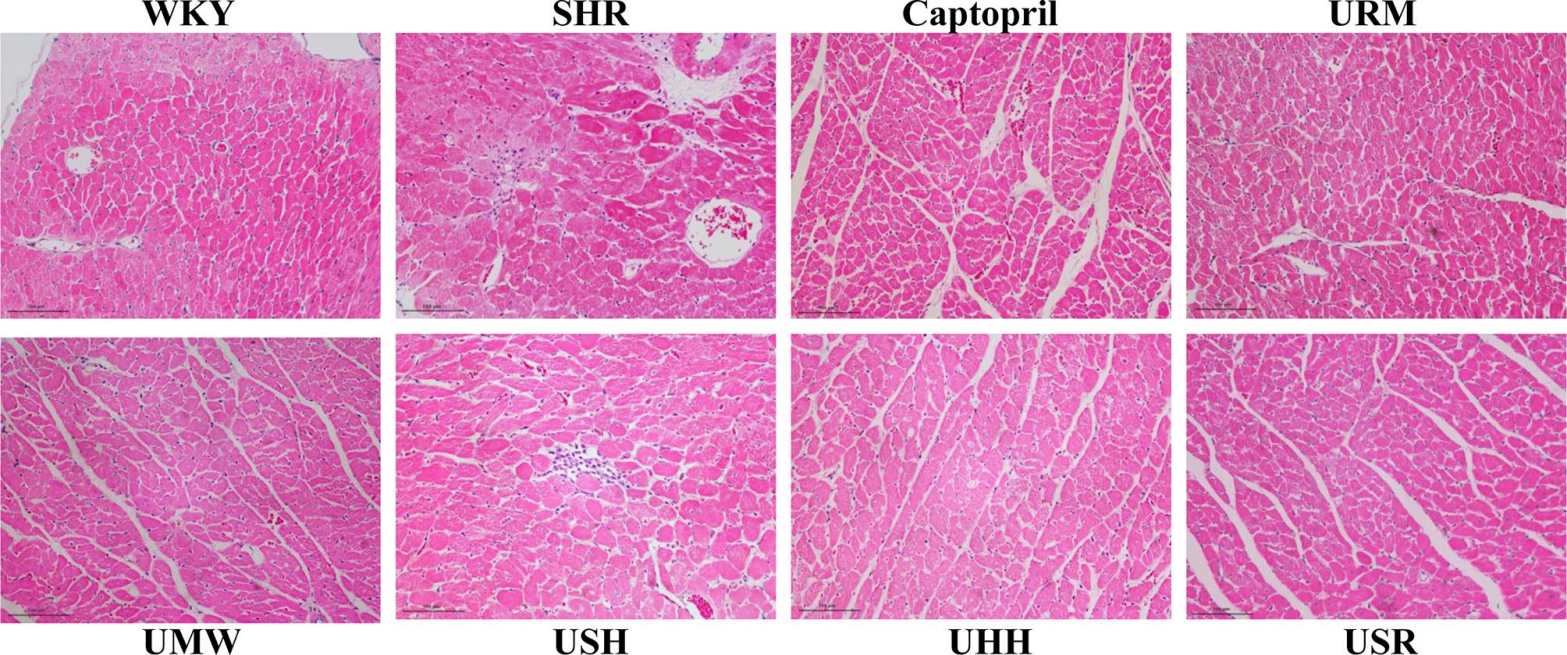
Treatment with UHs ameliorated cardiomycyte hypertrophy in spontaneously hypertensive rats (SHRs).

To assess the myocardial protection effects of the five different UHs, H&E staining was applied, and the results are shown in **Figure 3**. Cardiomyocyte hypertrophy was present as the increased cross-sectional size of myocytes in SHRs group, and UHs treatment qualitatively decreased the size of the cardiomyocytes compared to those of the SHRs.

### Metabolic Phenotype Changes in SHR After Treatment With Different UHs

**Figure 4:**
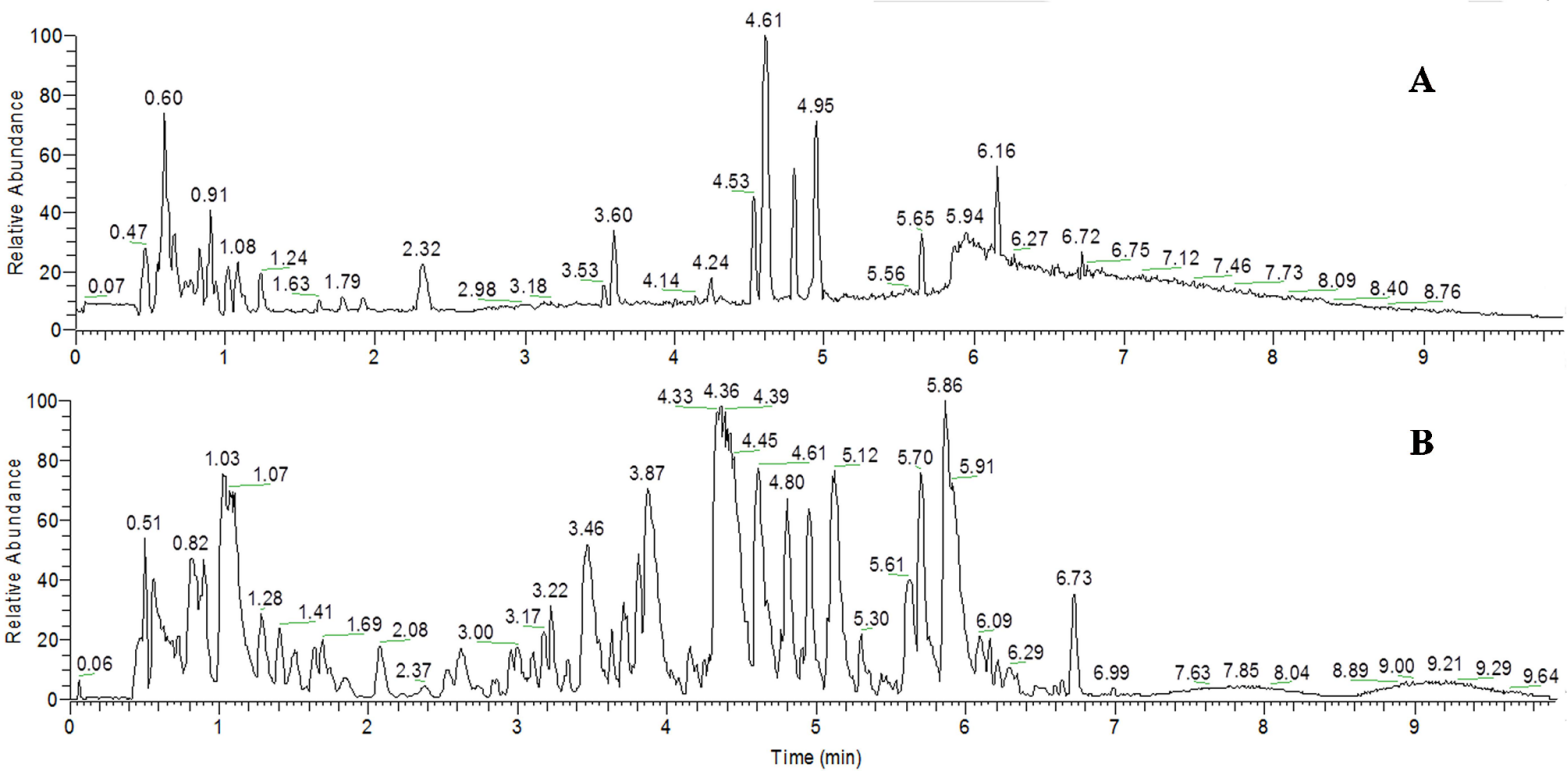
Representative base peak intensity chromatogram of the quality control (QC) samples obtained in the ESI positive mode **(A)** and negative mode **(B)** based on UHPLC/LTQ-Orbitrap-MS.

UHPLC-MS analysis under optimized condition simultaneously detected several different types of endogenous metabolites in a 12-min run. The typical base peak intensity (BPI) chromatograms of the urine samples in the positive and negative modes are shown in **Figure 4**.

**Figure 5:**
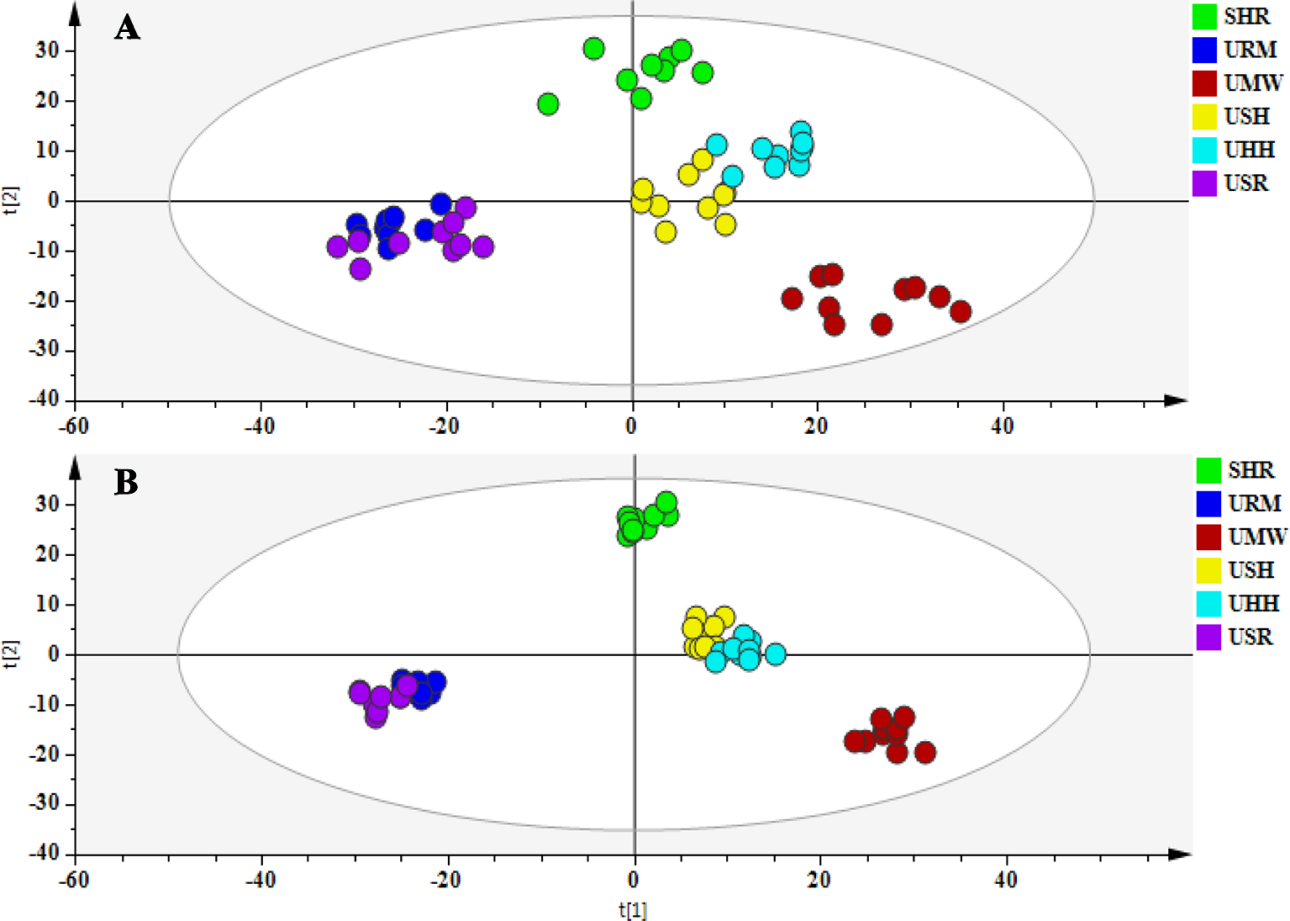
O2PLS-DA score plots of urine metabolic profiling of the SHR and treatment groups in the positive mode **(A)** and the negative mode **(B)**.

According to the O2PLS-DA algorithm, every dot represents a sample and contains information regarding all of the metabolites measured in the sample as well as their concentrations. The metabolites and their concentration determine the relative position of the sample in the scores plot. Therefore, the relative positions of the plots suggest the similarities and differences among the samples; the closer the dots, the more similar are the metabolite compositions and concentrations within the samples. Conversely, the further apart the dots, the greater the differences among the samples. To evaluate the overall changes of all of the endogenous metabolites in the different groups, O2PLS-DA approaches were used. The O2PLS-DA score plot (**Figure 5**) illustrated the distribution among the six groups. The URM group clustered close to the USR group, while the USH and the UHH groups displayed analogous metabolic phenotypes. However, the UMW-treated group was located far from the other groups, which indicated different metabolic changes had occurred compared with other treatment groups.

### Identification of Potential Biomarkers for Hypertension

To search for “potential biomarkers” for SBP, a newly established OPLS-DA model was employed to determine the changes in the endogenous metabolites between the WKY and SHR groups, based on their maximized intergroup differences and minimized intragroup differences. The parameters of the model (*R* ^2^ *Y* is 0.9931, *Q* ^2^ *Y* is 0.9635 in positive ion mode, *R* ^2^ *Y* is 0.9906, *Q* ^2^ *Y* is 0.9524 in negative ion mode) were acceptable, which indicated that the model had good abilities for prediction and reliability. After processing the OPLS-DA, variables with VIP values >1 were considered responsible for clustering and discrimination and were identified as potentially significant endogenous biomarkers. To select the “potential biomarkers” worthy of further study in the next step, these differential metabolites were validated using the Student’s *t* test and by correlation analysis (calculating the correlation between SBP and the response of the metabolites). The critical *p* value was set to 0.05 for significantly differential variables, and the correlation coefficient was set to 0.1 for an apparent correlation. Following these criteria, the qualified variable ions were identified by searching the MS and MS/MS fragments using the OSI/SMMS software and online free databases. Finally, 11, significantly differentiating, endogenous metabolites were identified, and eight endogenous metabolites were confirmed by comparison with commercial standards. The results of the identification experiments are shown in **Table 2**.

**Table 2:** Eleven identified potential biomarkers between the seven groups; (↑): upregulated, (↓): downregulated.

Mode	No.	tR(min)	m/z	Change						Identification	Pathway
				^a^SHR	^b^URM	^b^UMW	^b^UHH	^b^USH	^b^USR		
**ES+**	1	3.51	220.1177	↑	↓		↓	↓	↓	Pantothenic acid^s^	β-Alanine metabolism
	2	4.79	377.1454	↑	↓				↓	Riboflavin^s^	Riboflavin metabolism
	3	3.75	393.1680	↑	↓	↓	↓	↓	↓	Unknown	Unknown
**ES-**	4	3.29	131.0350	↓			↑	↑		2-Methylsuccinic acid^s^	Citric acid cycle
	5	3.75	145.0507	↓			↑	↑		2-Methylglutaric acid^s^	Unknown
	6	5.29	173.082	↓		↑	↑	↑		Suberic acid^p^	Fatty acid metabolism
	7	0.65	179.0562	↓		↑				Myoinositol^s^	Inositol phosphate metabolism
	8	3.98	181.0506	↑	↓				↓	Hydroxyphenyllactate^s^	Tyrosine metabolism
	9	2.58	182.0457	↓	↑		↑	↑	↑	4-Pyridoxic acid^s^	Vitamin B_6_ metabolism
	10	7.04	229.1442	↓		↑	↑	↑		Dodecanedioic aicd^s^	Fatty acid metabolism
	11	2.72	267.0732	↓	↑		↑	↑	↑	Inosine^s^	Purine metabolism

^a^Trends of the model group compared with the normal group of metabolites. ^b^Trends of the UHs groups compared with the model group of metabolites. ^s^Metabolites that were validated with authentic standards. ^P^Metabolites that were putatively annotated.

### Pathway Analysis of the SBP-Related Metabolites

To investigate the metabolic pathways and the specific compounds altered by the UHs treatment, compounds that displayed differently between SHRs and WKYs (**Table 2**) were identified using an online database of metabolic pathways (KEGG, http://www.genome.jp/kegg/). Eight related pathways were disturbed in SHRs (**Figure S1**, Supporting Information).

## Discussion

### Quality Assurance for the Metabonomics Study

In this study, strict quality control methods were established for each stage of the metabonomics assessment. First, the captopril-treated group, compared with the SHR control group, showed a decline in SBP during the 43 days of treatment. Using captopril, a classic antihypertensive drug, as the positive control for the effects on lowering high blood pressure, it showed that the SHR model was reliable for antihypertensive research and that it could be used for the subsequent metabonomics analysis of the five UHs. Second, the LC-MS analysis method and data processing workflow of the experimental samples were carefully evaluated using the quality control (QC) samples and the deuterated internal standards, respectively. This indicated that the established method and the workflow were both repeatable and reliable (**Table S1**, Supporting Information). These two procedures provided a strong foundation for the subsequent identification of the “potential biomarkers” and the pathway analysis in metabonomics. These metabonomics results indicate the different types of pathways which are disturbed by the hypertensive disease state and which of those could be affected by the five different UHs. This knowledge contributed to the elucidation of the underlying holistic effects and provided an explanation of the presumed mechanism(s) of the UHs as they depress SBP.

### Hypotensive Effect and Regulatory Effect of Five Different UHs

The hypotensive effects of the five different UHs, which are indicated in the Chinese Pharmacopeia (2015 Edition), were studied in a spontaneously hypertensive rat (SHR) model for 6 weeks. Compared with the SHR group, as shown in **Figure 2**, the SBP decreased rapidly in the five UHs groups after the first treatment with URM, UMW, UHH, USH, and USR and maintained the low SBP characteristics over the course of the treatment. At each time point, the individual UHs-treated groups had similar SBP values, with no significant differences observed. These results suggest that the five UHs displayed the same hypotensive activity at the same dose.

As illustrated by the O2PLS-DA score plot (**Figure S2**, Supporting Information), the SHR plots were distributed in the left side of the figure and are separated from the WKY plots. This indicates that the metabolomic variations may result from the development of hypertension. Furthermore, all of the samples from the five UHs and captopril groups were close to the SHR group and further away from the WKY group after 6 weeks of treatment. These results suggest that a difference exists between the treatment groups and the WKY group in terms of their metabolic phenotypes. This also infers that the hypotensive effects of the five different UHs were not directly related to the overall regulation of metabolism in the treatment groups. This result is different compared with the antihypertensive effects of the total ginsenosides. In that instance, after treatment, the metabolomics phenotypes of total ginsenosides group shifted towards the WKY group (AA et al., 2010).

### Comparative Regulatory Effects of URM, UMW, USH, UHH, and USR on the Metabolic Phenotype

The results discussed above show that the five different UHs had analogous antihypertensive effects. However, as shown in **Figure 5**, there were different metabolic phenotypes among the five UHs treatment groups. Therefore, the different chemical constituents in the five species of UHs effected regulation of the metabolism. As indicated by the O2PLS-DA score plot, a metabolomic similarity was revealed between the URM and the USR groups. Meanwhile, the USH and UHH samples also probably had an analogous metabolic phenotype. The UMW-treated group was showed to have a very different regulatory effect on the metabonomic profile. Since there were significant differences concerning the main metabolites of the five different UHs (as shown in **Figure 1**), these results suggest that corynoxine B and corynoxine have a strong regulatory effect on metabolism but that hirsuteine, hirsutine, uncarine B, and uncarine A provide only a weak regulatory effect.

**Figure 6:**
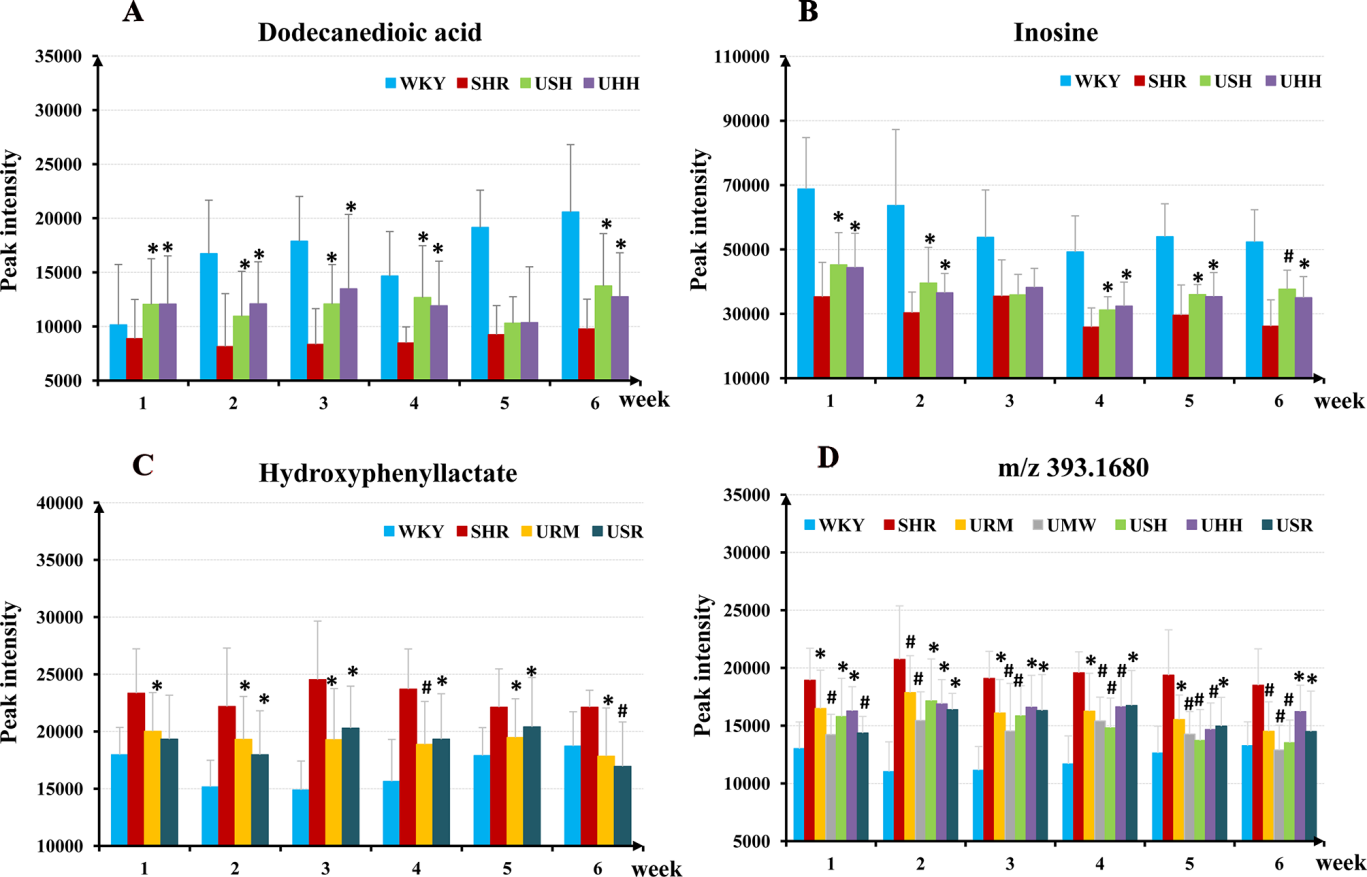
The relative abundances of the dodecanedioic acid **(A)**, inosine **(B)**, hydroxyphenyllactate **(C)**, and the potential hypertension biomarker (*m*/*z* 393.1680) **(D)** in the WKY group, the SHR group, and the different treatment groups within the 6-week intervention period. *p < 0.05, ^#^ *p* < 0.01 compared to the SHR group.

To investigate the specific compounds regulated by the UHs intervention, the compounds that exhibited differential variation in SHRs and WKYs were designated. In total, 11 compounds were identified that differed significantly between the SHRs and WKYs (**Table 2**). These compounds were regulated at different levels in the five species of UHs. Treatment with USH and UHH led to restoration of 8 of the 11 discriminatory metabolites toward normal levels. URM and USR adjusted the levels of six metabolites toward normal values, and the levels of four metabolites were adjusted after treatment with UMW. The regulatory effects of these UHs were also reflected in the diverse regulation of specific compounds. For example, USH and UHH adjusted the level of dodecanedioic acid and inosine to normal levels, which were significantly different from the level in SHRs after administration from the first to the sixth week (**Figures 6A, B**). In contrast, the other three UHs did not significantly regulate these two metabolites during the course of the 6 weeks administration. Interestingly, URM and USR regulated hydroxyphenyllactate to normal levels during the whole administration period (**Figure 6C**).

### Relationship Between SBP and Potential Hypertension Biomarkers

The results discussed above show that each of the five species of UHs had an apparent antihypertensive effect. The indole alkaloids were the main active constituents in the five species of UHs, supporting the hypothesis that a similar antihypertensive mechanism was possessed by the five different UHs. After treatment with the UHs, the SBP decreased rapidly in the five UHs groups and maintained approximately relatively low SBP characteristics as the 6-week treatment continued. These results suggest that the potential biomarkers for hypertension in the treatment groups were restored toward those in the normotensive control by the first administration of the UHs and were kept normal in the subsequent treatment regimen. In order to search for potential hypertension biomarkers, compounds were selected that exhibited differential variation between the SHRs and the WKYs. One compound, with *m*/*z* 393.1680 (+ve ion mode), was restored to normal values in five species of UHs groups as soon as administration was initiated (**Figure 6D**). The results indicate that this compound shows a correlation with SBP lowering.

It was important to determine whether there is a statistical relationship between the SBP and the potential hypertension biomarker. Linear regression between the SBP values and the peak intensity of the metabolites revealed that this compound correlated with the SBP (**Figure S3**, Supporting Information). This observation suggests that the compound is a potential biomarker of hypertension and a quantitative biomarker of the therapeutic response to the five species of UHs. Further tests are needed to verify the alteration of *m*/*z* 393.1680 to UHs at various doses and treatment lengths.

## Conclusions

UHs from five different plant origins have shown similar antihypertensive effects *in vivo* in spite of their very different chromatographic fingerprints. A metabonomics study based on LTQ-Orbitrap MS analysis and combined with a “dynamic analysis” strategy was successfully established to investigate the metabolic phenotype of UHs-treated SHR. The study revealed the metabolic perturbation characteristics of SHRs with UHs treatments, which indicated a weak regulatory effect of UHs on the metabolic disorder. In addition, the regulatory effects of the five species of UHs on the metabolic phenotype were different. These results may provide useful information in understanding the possible antihypertensive mechanisms of the different UHs. A similar antihypertensive effect could be produced by the five different UHs, whereas this similarity might be circumstantial and caused by different metabolic pathways due to their different indole alkaloid constituents. The results in five different UHs are in consistent with the “different species, same effect” phenomenon, and our study provides a promising strategy to elucidate the underlying mechanisms of this phenomenon in TCMs. Finally, a common, potential hypertension biomarker for all five different UHs was discovered. This biomarker might be used in the further as an endogenous efficacy index for clinically evaluating the antihypertensive effect of the UHs from different botanical origins.

## Data Availability

Publicly available datasets were analyzed in this study. This data can be found here: http://www.genome.jp/kegg/.

## Ethics Statement

This study was carried out in accordance with the recommendations of guide for the care and use of laboratory animals, the Animal Ethics Committee of Shanghai Institute of Materia Medica (Shanghai, China). The protocol was approved by the Animal Ethics Committee of Shanghai Institute of Materia Medica (Shanghai, China).

## Author Contributions

D-AG, WaW, and JH were responsible for the conception and design of the study. ZF analyzed the data and drafted the paper. YY, YD, WeW, and XW contributed to the data collection and image processing. HZ and LZ took part in the discussion in the paper. All authors read and approved the final manuscript.

## Conflict of Interest Statement

The authors declare that the research was conducted in the absence of any commercial or financial relationships that could be construed as a potential conflict of interest.
