# Supplementary material for: Dissecting the Metabolic Phenotype of the Antihypertensive Effects of Five Uncaria Species on Spontaneously Hypertensive Rats
Source: Front Pharmacol. 2019 Jul 30;10:845. doi: 10.3389/fphar.2019.00845 (PMC6682664; doi:10.3389/fphar.2019.00845)
Supplement: Supplementary file 1 [file DataSheet_1.docx]

**Chromatography and mass spectrometry conditions of sample analysis**

High-resolution mass spectra of five different water extract were obtained on a LTQ-Orbitrap Velos Pro hybrid mass spectrometer(Thermo Fisher Scientific, San Jose, CA, USA) connected to an Ultimate 3000 UHPLC system. The separation of samples was performed on a Waters ACQUITY UPLC BEH C_18_ column(1.7 µm, 2.1mm×100mm) with an online filter. The mobile phase comprised solvent A:100 % H_2_O(0.1% ammonia) and solvent B:100% acetonitrile, with gradient elution as follows: 10-20% B at 0-8min, 20-26% B at 8--13min, 26-31% B at 13-16min, 31-34% B at 16-18min, 34-41% B at 18-19min, 41-46% B at 19-26min, 46-50% B at 26-30min, 50-90% B at 30-32min, 90% B at 32-35min. The flow rate was kept at 0.40 mL/min. The temperatures of autosampler and column were kept at 4℃ and 30℃, respectively. The injection volume of all samples was set at 1.0 µL. Positive ion detection mode were conducted in this analysis. The ESI source parameters were set as follows: ion spray voltage 3.8 kV, capillary temperature 350℃, source heater temperature 300℃, sheath gas (N_2_) 40 arbitrary units, auxiliary gas (N_2_) 10 arbitrary units, and sweep gas(N_2_) 0 arbitrary units. The Orbitrap analyzer scanned the mass with the range of m/z 150-1000 and a resolution of 30000 for MS and 7500 for MS^n^(n=3). A normalized collision energy(NCE) of 35% was used. Dynamic exclusion function was enabled, and parameters were set at follow: repeat count, 5; repeat duration, 10s; exclusion list size, 50; exclusion duration, 20s.

**Table S1 The repeatability and stability data of the proposed method**

| Mode | Selected m/z | Rt(min) | |  | Peak area | |
| --- | --- | --- | --- | --- | --- | --- |
|  |  | Mean | RSD% |  | Mean | RSD% |
| ES^+^ | 114.0659 | 0.60 | 0.52% |  | 17198367 | 5.78% |
|  | 169.0358 | 1.09 | 0.24% |  | 5822588 | 9.51% |
|  | 245.1609 | 2.33 | 0.40% |  | 12041275 | 4.59% |
|  | 297.1450 | 3.58 | 0.30% |  | 9635513 | 8.21% |
|  | 338.0866 | 4.52 | 0.19% |  | 9921531 | 7.88% |
|  | 340.1020 | 4.79 | 0.15% |  | 12116966 | 2.75% |
|  | 194.0809 | 4.94 | 0.13% |  | 19986011 | 4.65% |
|  | 170.0603 | 5.65 | 0.09% |  | 8502524 | 3.61% |
|  | 255.0644 | 6.15 | 0.10% |  | 13946476 | 5.23% |
|  | 271.0597 | 6.72 | 0.13% |  | 7319360 | 7.64% |
| ES^-^ | 117.0193 | 1.40 | 0.27% |  | 8655737 | 5.04% |
|  | 204.9812 | 2.07 | 0.54% |  | 10392956 | 4.67% |
|  | 215.1033 | 2.60 | 0.47% |  | 14124887 | 3.60% |
|  | 227.9969 | 3.22 | 0.41% |  | 13797414 | 7.49% |
|  | 261.0075 | 3.82 | 0.70% |  | 25121706 | 7.09% |
|  | 242.0132 | 4.82 | 0.26% |  | 48030276 | 1.51% |
|  | 192.0665 | 4.94 | 0.08% |  | 33565079 | 1.63% |
|  | 283.0820 | 5.11 | 0.13% |  | 29568682 | 3.51% |
|  | 297.0981 | 5.69 | 0.08% |  | 43639941 | 4.59% |
|  | 269.0457 | 6.72 | 0.07% |  | 15898527 | 9.19% |


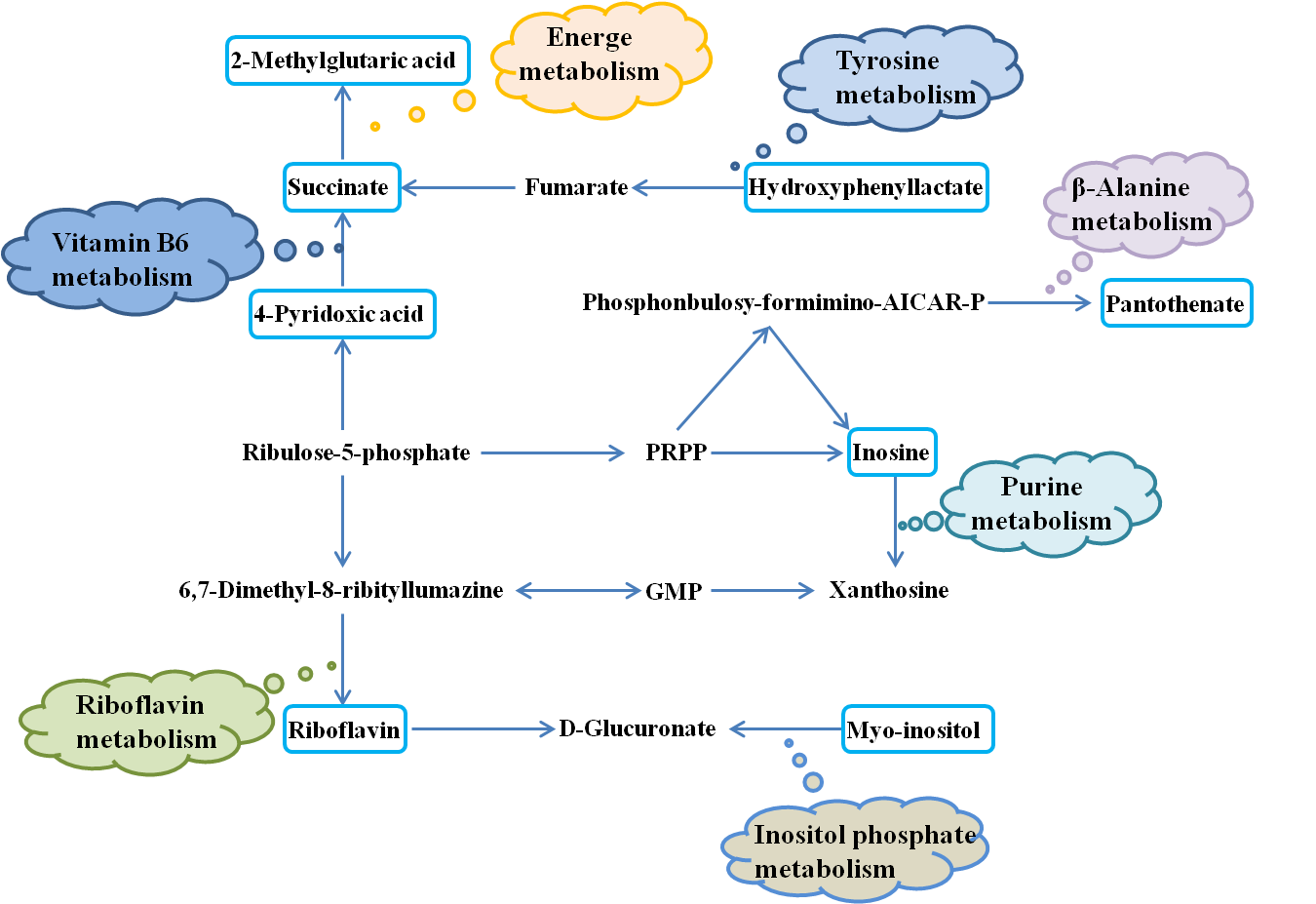


**Figure.S1 The network of the 8 identified biomarkers according to the KEGG Pathway Database. The solid line area denotes the identified biomarkers.**


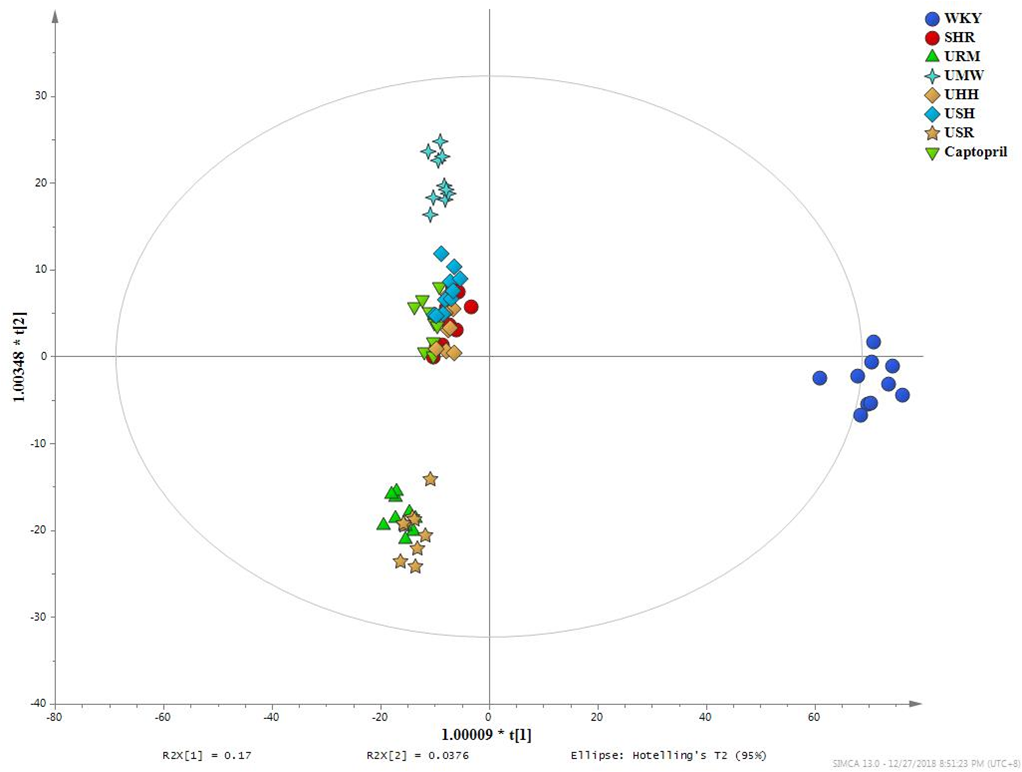


**Figure.S2 O2PLS-DA score plots of urine metabolic profiling.**

**
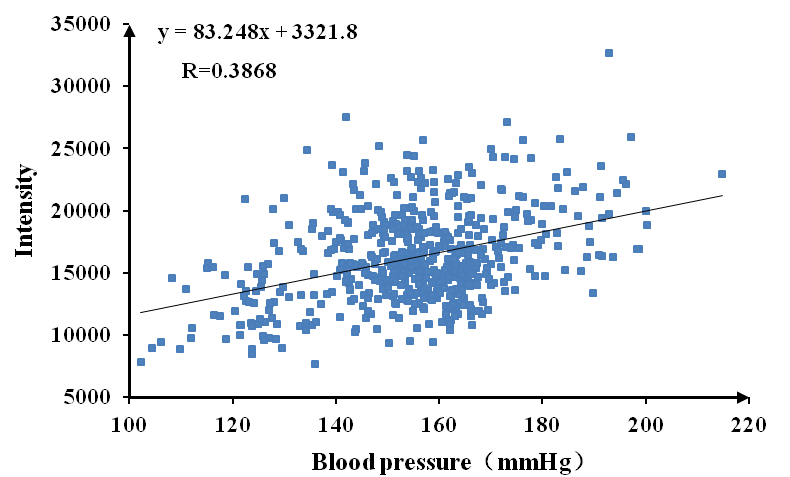
**

**Figure.S3 Linear regression profiles of BP and potential markers of hypertension. Data included all of the experimental groups treated by captopril, URM,UMW, USH, UHH, USR, the SHR control and the WKY control.**
